# Supplementary material for: GS-2: A Novel Broad-Spectrum Agent for Environmental Microbial Control
Source: Biomolecules. 2022 Sep 13;12(9):1293. doi: 10.3390/biom12091293 (PMC9496126; doi:10.3390/biom12091293)
Supplement: Supplementary file 1 [file biomolecules-12-01293-s001.zip › biomolecules-1896965 fc done - supplementary.pdf]

biomolecules

MDPI

A

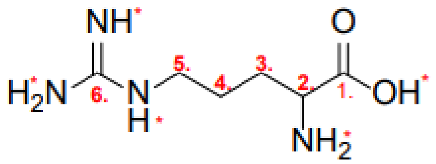

B

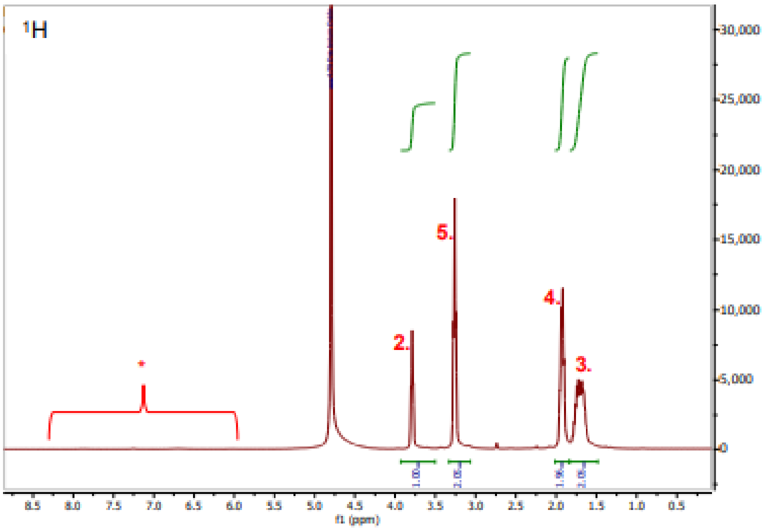

¹H NMR analysis		
Position	Expected number of protons	Found number of protons
2	1	1
3	2	2.05
4	2	1.96
5	2	2.05

C

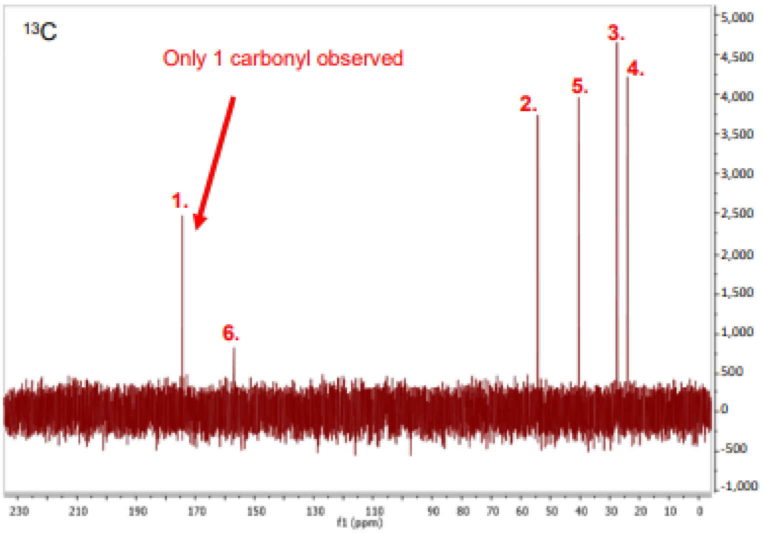

Supplementary Figure S1. NMR analysis of aqueous fraction of GS-2. (**A**) Schematic representation of L-arginine with carbons labeled 1–6. *These protons underrepresented in NMR plots due to fast chemical exchange. (**B**) ^{1}H and (**C**) ^{13}C NMR analysis of the aqueous fraction of GS-2.

A

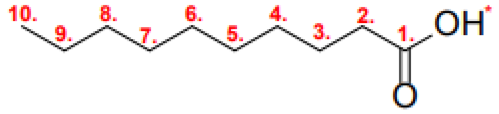

B

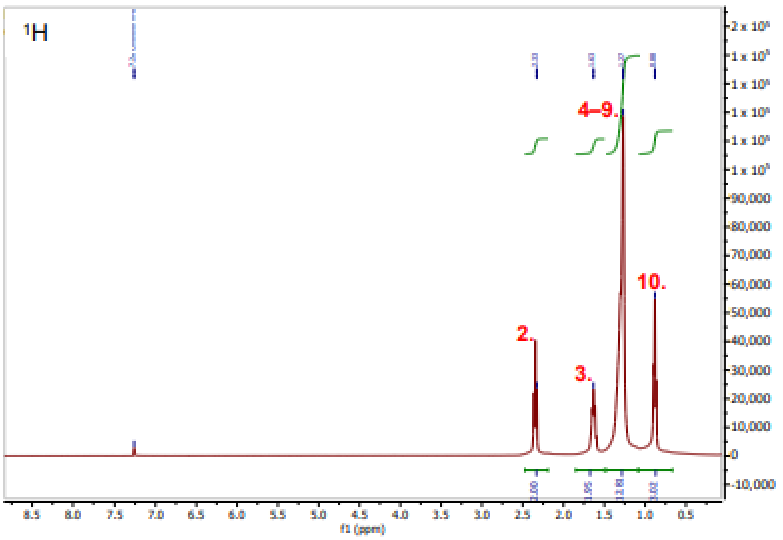

^{1}H NMR analysis		
Position	Expected number of protons	Found number of protons
2	2	2.0
3	2	1.95
4–9	12	12.81
10	3	3.02

C

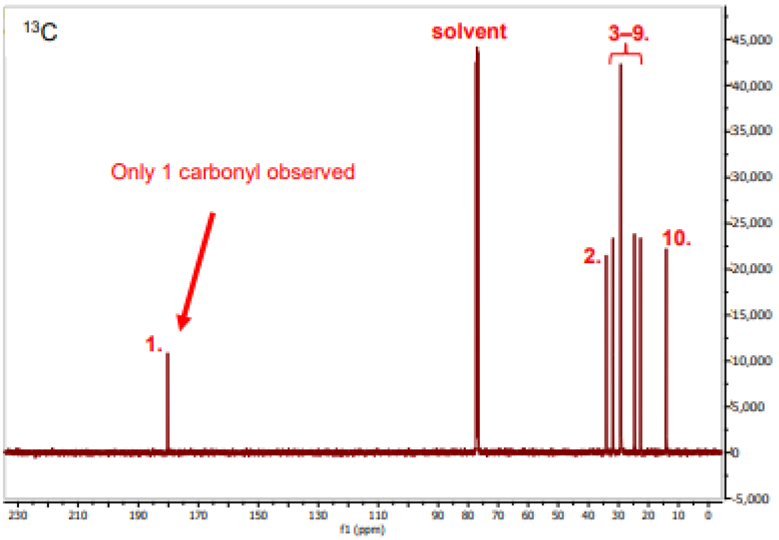

Supplementary Figure S2. NMR analysis of heptane fraction of GS-2. (**A**) Schematic representation of capric acid with carbons labeled 1–10. *These protons are underrepresented in NMR plots due to fast chemical exchange. (**B**) ^{1}H and (**C**) ^{13}C NMR analysis of the heptane fraction of GS-2.

A

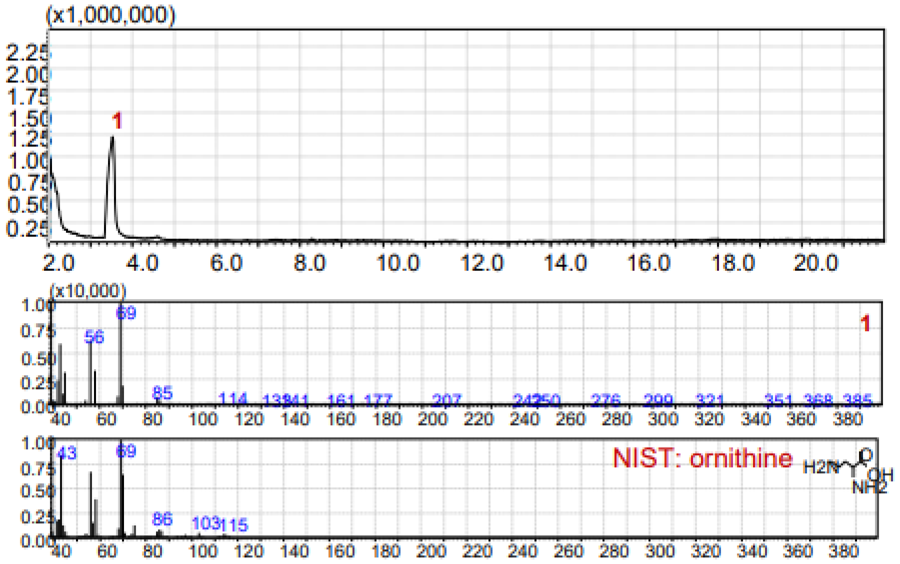

B

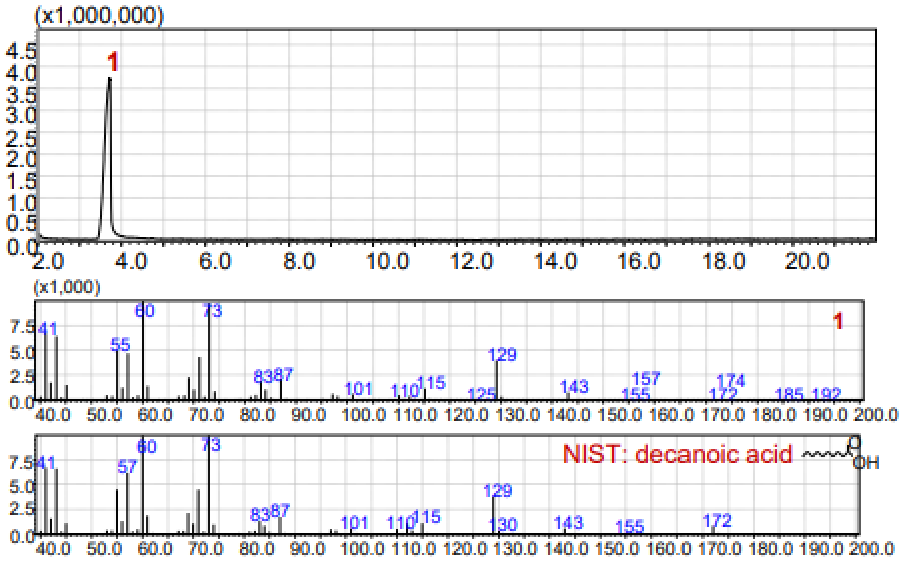

Supplementary Figure S3. GCMS analysis of GS-2. (**A**) aqueous fraction and (**B**) heptane fraction of GS-2.
